# Combination of Polymeric Superplasticizers, Water Repellents and Pozzolanic Agents to Improve Air Lime-Based Grouts for Historic Masonry Repair

**DOI:** 10.3390/polym12040887

**Published:** 2020-04-11

**Authors:** Jesús Fidel González-Sánchez, Burcu Taşcı, José María Fernández, Íñigo Navarro-Blasco, José Ignacio Alvarez

**Affiliations:** 1 MATCH Research Group, Chemistry Department, School of Sciences, University of Navarra, 31008 Pamplona, Spain; jgonzalez.65@alumni.unav.es (J.F.G.-S.); jmfdez@unav.es (J.M.F.); inavarro@unav.es (Í.N.-B.); 2 Department of Architecture, Izmir Katip Çelebi University, 35620 Izmir, Turkey; burcutasci@iyte.edu.tr

**Keywords:** polymeric superplasticizers, zeta potential, adsorption isotherms, steric hindrance, grouts, injectability, hydrorepellency, freeze–thaw cycles

## Abstract

This paper presents the experimental procedure to develop air lime-based injection grouts, including polymeric superplasticizers, a water repellent agent and pozzolanic agents as additives. Our research focuses on the development of grouts to improve various characteristics simultaneously by combining different additions and admixtures. Aiming to improve the injectability of the grouts, in this study, different polymeric superplasticizers were added, namely polycarboxylated-ether derivative (PCE), polynaphthalene sulfonate (PNS) and condensate of melamine-formaldehyde sulfonate (SMFC). As a water-repellent agent, sodium oleate was used to reduce the water absorption. The enhancement of the strength and setting time was intended by using microsilica and metakaolin as pozzolanic mineral additions. Compatibility between the different admixtures and action mechanism of the different polymers were studied by means of zeta potential and adsorption isotherms measurements. Diverse grout mixtures were produced and investigated by assessing their injectability, fluidity, stability, compressive strength, hydrophobicity and durability. This research led to several suitable mixtures produced by using more than one component, to enhance efficiency and to provide better performance of grouts. According to the results, the grout composed of air lime, metakaolin, sodium oleate and PCE was found to be the most effective composition, improving the mechanical strength, injectability and hydrophobicity.

## 1. Introduction

One of the most widely used methods addressed to repair different masonry defects and cavities in the preservation of the Built Heritage is the injection of grouts [1,2,3]. Grouts, fluid mixtures made of water, binder and additives, must properly flow—under an appropriate pressure—into a masonry wall in a fresh state [4,5]. The literature has pointed out some requirements for grouts in fresh state, such as high penetrability (i.e., injectability) and good stability of the suspension (meaning no, or at least limited, segregation and bleeding) [6,7]. In addition, the grout must be chemically compatible with the ancient masonry, in order to prevent the historic structure from damages caused, for example, by high contents in soluble salts. Mechanical compatibility is another requirement; for instance, repair materials with too-high stiffness are not compatible with the old masonry [8,9].

Taking into account these aspects, natural hydraulic lime (NHL) and hydraulic lime (as obtained by air lime with pozzolana) have been the most widely used binders for repair grouts of the Architectural Heritage, as they offer suitable chemical and mechanical compatibility [1,3,4,6,7,9,10,11,12,13,14]. Pure air lime grouts face up to the poor water retention, excessive drying and subsequent shrinkage [9,15] and have been mainly tested for non-structural applications [14], whereas cement-based grouts or organic grouts are not chemically compatible, and excessive stiffness is also observed for the former [16].

Therefore, one of the main challenges concerning the research on these materials is the design of tailored grouts [4]. Additives and admixtures are very useful to enhance different properties of the grouts: For example, polymers behaving as superplasticizers would promote injectability, as a critical parameter for the applicability of the grouts, which will also improve due to the mixing water reduction in the final hardened microstructure [11,12,14,17]; water-repellent agents would impart hydrophobicity to the hardened grouts, reducing the water uptake [18,19,20]; and pozzolans additions would increase the mechanical resistance and accelerate the setting time [11,15,21,22,23].

Some previous works have highlighted the advantages of these additives/admixtures, individually added to binding materials. For example, various advantages of using polymers acting as superplasticizers (SPs) in lime grouts have been ascertained [6,7,10,11,12,13,14,17,22,23,24,25]. As polymeric admixtures, the superplasticizers increase the fluidity of the fresh grouts, promote suitable injectability and improve the workability at a constant water/binder ratio. When these polymers are added into the grout mixture, they prevent particles from agglomeration acting as dispersing agents and thus reducing the water demand [21,26]. Most works have addressed lime-based grouts with the addition of a superplasticizer (commonly polycarboxylated ether) [11,12,14,17,23,25,27]. Much more limited information has been produced on the effect in lime grouts of other SPs such as polynaphthalene sulfonate (PNS) and poly-melamine sulfonate (SMFC), commonly tested in the cement chemistry [24,25,28].

The use of water-repellents is of importance to minimize the uptake of water in grouts and mortars. The access of water to the inner part of hardened grouts and mortars is largely detrimental for the structural integrity of the masonry: Water dissolves soluble salts, giving rise to efflorescences. Furthermore, it takes part in freeze–thaw cycles, provoking severe mechanical damages to the masonry. Hydrophobicity imparted by water-repellent admixtures would enhance the long-term resistance of the grouts. Dual organic compounds, with a polar moiety (usually a carboxylic group) and a hydrophobic tail, such as calcium stearate and calcium oleate, have been studied [18,19,20].

The use of pozzolans has been widely reported, and some studies have described the possibility of improving resistance, accelerating setting times and permitting hardening—even when CO_2_ is scarcely available—by adding pozzolana to mixtures [29], which is noteworthy for injection grouts applied in deep fissures and cavities with restricted CO_2_ access. Metakaolin (MK) has been one of the most widespread studied pozzolans, although nanosilica has also been the target in some research works [21,30,31,32]. MK is usually processed by calcination of high-purity kaolin clay at temperatures ranging between 650 and 800 °C. It contains silica and alumina in an active form, and they react with the calcium hydroxide of the air lime (Ca(OH)_2_, CH), yielding hydrated calcium silicate (C-S-H) phases, and also C_2_ASH_8_ and C_4_AH_13_ as hydrated silico-aluminate and hydrated aluminate phases, respectively. The filler effect of metakaolin, together with the production of new hydrated phases, results in improved air lime-based grouts’ properties, such as setting time and compressive strength, while also preventing hardened grouts from microcracking [21]. The effect of the increasing replacement of NHL by metakaolin has also been studied [15]. Another pozzolana tested in binding materials is microsilica (MS), generally comprised of amorphous silicon dioxide as a fine powder. The material is a product of the silicon and ferrosilicon, and it is produced in smelting industries. Studies with MS in concrete showed favorable results for strength-supporting sulphate exposure [33,34].

A promising way of modulating the characteristics of the grouts is the simultaneous combination of different admixtures and mineral additions. Only a few works have dealt with the obtainment of grouts by combining, for example, a superplasticizer and a water retainer [9,16,21], or a superplasticizer together with a pozzolana (metakaolin, nanosilica or silica fume) [17,25], but a systematic study on quaternary mixtures, analyzing the effect of air lime as binder with polymeric superplasticizers, a water-repellent agent and pozzolanic additions, is not available.

Accordingly, the context and the rationale of the current work is that synergistic simultaneous combinations between air lime, a superplasticizer, a water-repellent agent and pozzolana would make it possible to obtain tailored injection grouts suitable for restoration of the Built Heritage.

The following raw materials were used for the combinations: calcitic air lime, three different polymer-based admixtures, which are superplasticizers: polycarboxylate ether (PCE), polynaphthalene sulfonate (PNS) and poly-melamine sulfonate (SMFC); a water-repellent agent (sodium oleate); and two types of pozzolanic addition (metakaolin and microsilica). Fresh state properties of the grouts, such as injectability, bleeding and fluidity (as measured by the slump test), were determined. Action mechanisms, interactions and compatibility between the tested admixtures were assessed by measuring zeta potential of the suspensions and adsorption isotherms of the admixtures. Hardened state was also assessed, by evaluating the compressive strengths, carbonation rate, hydrophobicity and pore structure, and the durability of the grouts was finally studied by exposing the samples to freezing-thawing cycles. The influence of the different additives/admixtures of the grout compositions on these parameters is later discussed.

## 2. Materials and Methods 

### 2.1. Materials and Composition of the Grouts

Mixing proportion of the grouts was 1:3 binder/aggregate weight ratio, according to previous prescriptions [1]. Binder was CL-90 hydrated calcitic lime (Cal Industrial S.A. Navarra, Spain) (CaO percentage 68.53%, with major impurities of MgO (3.29%), SO_3_ (1.37%) and SiO_2_ (1.03%)). Mean particle size was 10 μm (less than 10% > 50 μm). A very fine limestone aggregate, with particle size lower than 2 mm, was used and supplied by CTH (Huarte, Navarra, Spain). 

For the different mixtures, the following components were added, with respect to lime, when necessary:

- Polymer-based superplasticizer (SP) (two different dosages 0.5% and 1% by weight of lime (bwol)): polycarboxylate ether (PCE), commercialized by BASF as Melflux; condensate of melamine-formaldehyde sulfonate (SMFC), commercialized by BASF as Melment F10 (Ludwigshafen, Germany); polynaphthalene sulfonate (PNS), commercialized by FOSROC International as Conplast SP340 Fa (Fosroc Euco S.A., Izurtza, Spain).
- Water-repellent agent (0.5% bwol): sodium oleate (O), provided as a commercial product: HISA A 2388 N by ADI-Center-S.L.U (Barcelona, Spain).
- Pozzolanic additions (20% bwol): Metakaolin (MK) (Metaver, supplied by, NEWCHEM, Pfäffikon, Switzerland) and microsilica (MS), supplied by ULMEN Europa (Castellón, Spain).

**Figure 1:**
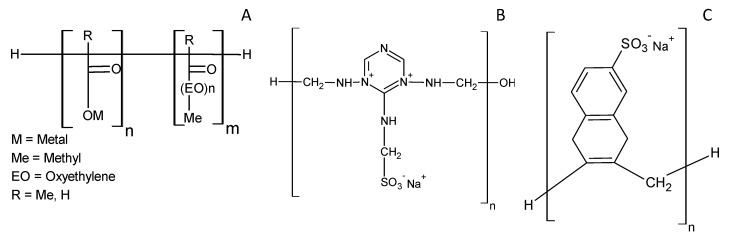
Structures of different superplasticizer: (**A**) PCE, (**B**) SMFC and (**C**) PNS.

The first polymer-based superplasticizer used was PCE (Figure 1A), which consists of one main linear backbone with side carboxylate and ether groups. The carboxylate groups are the anchoring groups by which the adsorption of these admixtures to cement particles takes place [17,26].

The second polymeric SP belongs to the family of the sulfonated melamine formaldehyde condensates (SMFC) (Figure 1B). In this synthetic polymer, each repeating unit contains one sulfonate group. The condensation number (n) is usually in the 50–60 range, giving a molecular weight in the order of 12,000–15,000 [35].

The third employed polymer was PNS (Figure 1C), in which its molecular structure is characterized by a hydrophobic moiety (naphthalene) and a hydrophilic part (sulfonate groups).

In some previous works, the most relevant properties of these polymers, such as molecular weight, anionic charge density and elemental composition, as well as the methods to assess these values, were reported [11,27]. The molecular weights (Mw) of the polymers were determined by size-exclusion chromatography. Anionic charge densities of each one of these polyelectrolytes were obtained by titration, using the positively charged Poly-DADMAC (acid-base titration for oleate). A LECO analyzer (LECO Corporation, St Joseph, MI, USA) was used to determine the elemental composition of the polymers. Table 1 gathers these values.

Sodium oleate was added as a water-repellent agent (O) (see characteristics in Table 1). This compound is characterized by a long non-polar hydrocarbon chain and a polar carboxylate group at one end, having a bipolar nature. Therefore, it may be adsorbed and concentrate at the air–paste interface, usually in the air bubble surface. This fact causes reinforcement of the air bubbles and avoids coalescence [27]. This admixture has also been reported in the scientific literature on cement mortars as AEAs [29,30,31,32]. The dosage was 0.5% of the total dried mortar weight, in agreement with a previous work that reported the enhancement lime-based mortars at that dosage [27].

**Figure 2:**
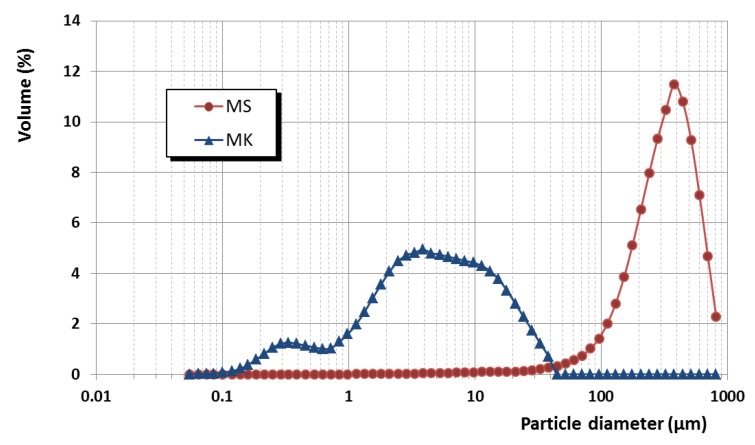
Particle size distribution of the pozzolans.

Specific surface areas, as measured by the BET method after N_2_ adsorption isotherms (ASAP 2020, Micromeritics, Norcross, GA, USA), for MK and MS were of 20.00 and 15.70 m^2^·g^−1^, respectively. According to the supplier, microsilica particles are spherical, main range of primary particle sizes between 0.2 and 1 µm, and the MS composition is at least 85% SiO_2_ content, with low carbon content [36]. The average particle sizes in aqueous suspensions were of ca. 3.9 µm for MK and 380 µm for MS (particle size distribution determined by laser diffraction in a Malvern Mastersizer, Malvern Instruments, Ltd., Malvern, UK, depicted in Figure 2), evidencing a clear agglomeration of the MS in comparison with the particle size of the primary particles [11,36].

With the aim of assessing the effect of the different admixtures in the properties of grouts, particularly in the injectability, a 31% of mixing water was established for all samples. This value was reached after carrying out an adjustment of the water demand of the control mortar (additives/admixtures-free) to obtain a spread flow diameter of 185 mm as measured in the flow table test. The different compositions of the 24 prepared and tested mixes are collected in Table 2.

### 2.2. Preparation Procedure and Curing Conditions

The grouts were prepared mixing the powdered hydrated calcitic lime, the sand and, when necessary, the pozzolanic addition and the solid admixtures (water-repellent and superplasticizers) for 5 min, using a solid-admixtures mixer BL-8-CA (Lleal, S.A., Spain). After this step, the mixing water was added and mixed for 90 s, at low speed, and adjusted according to UNE-EN 196-1, in a Proeti ETI 26.0072 (Proeti, Madrid, Spain) mixer [37]. 

Prismatic molds of 40 × 40 × 160 mm were used for casting fresh grouts. Standard EN 196-1 was followed for the filling in two layers and for the compaction using an automatic compactor (IBERTEST iB32-045E-1, S.A.E. Ibertest, Madrid, Spain), with the aim of removing the air bubbles present in the mixture. Molds were stored at lab conditions (20 °C and 60% RH), and hardened grouts were demolded 5 days later. Hardened state properties were studied after different curing ages: 7, 28, 91, 182 and 365 days. Representativeness of the results was guaranteed by testing three replicates of the grouts per each curing time.

### 2.3. Fresh-State Tests and Analyses

For all the following tests, at least three replicates were carried out for each one of the performed tests, and each one of the grouts’ compositions, so that the depicted values are an average value of all the recorded measurements.

- The flow table test (according to the EN 1015-3 [38]) was followed, to monitor the slump flow measurements, after 15 strokes of the flow table. The larger the spread diameter, the higher the fluidity of the grout.
- Workability was determined as the period in which the degree of stiffness of the grout hinders the penetration of a piston. Workability can be related to the setting time of the grouting mixture (the shorter the workability time, the shorter the setting time). According to the standard EN 1015-9 [39], every 15 min, a probe was slowly introduced into the fresh grout, scoring the weight, which was gradually increasing due to the hardening of the grout. When this weight reached 1500 g, the assay was concluded.
- A Zeta potential electroacoustic analyzer (ZetaProbe Analyzer, Colloidal Dynamics, Ponte Vedra Beach, FL, USA) was used to determine the surface charge of the suspensions of the air lime with the additives. Two batches of experiments were carried out:
  (a) Initial media of air lime, water and, when necessary, pozzolanic additives and sodium oleate were prepared by following the same compositions detailed in Table 2. Solutions of polymer-based superplasticizers (1% w/w) were then used as titrant media, and zeta potential values were continuously monitored.
  (b) Initial media of air lime, water and, when necessary, pozzolanic additives and SP were prepared by following the same compositions detailed in Table 2. Solution of sodium oleate (1% w/w) was, in this case, used as titrant media, monitoring the zeta potential values.
- Adsorption isotherms were obtained after carrying out different sorption assays. Different batches of flasks were prepared: one, with 5 g of air lime per 25 mL of water; two more batches with also pozzolanic additive (either MS or MK, 20 wt.% with respect to the lime). In some flasks, when required, pre-adsorption of some admixtures was also carried out incorporating either SP or oleate (1 wt.% or 0.5 wt.% with respect to the lime, according to the proportions reported in Table 2) and mixing the dispersions for 30 min. The adsorption of the admixtures, either sodium oleate or SPs, was studied adding increasing amounts of the admixture (0.0125, 0.0250, 0.0375, 0.0500, 0.1000, 0.1500, 0.2000 g) to the different flasks. Dispersions were magnetically stirred for 30 min and then centrifuged at 8000× *g* for 15 min. The supernatant was collected and filtered (0.45 µm PTFE filters). The difference between the initial (added) and final concentration (remaining solution concentration) was deemed to be the admixture adsorbed amount. UV–VIS spectrophotometry was used to measure the concentration of the admixture in the solution (maxima at λ = 221, 222 and 296 nm for PCE, SMFC and PNS). The mathematical fitting of the adsorption data was calculated for Langmuir and Freundlich models.
- Bleeding test refers to the determination of a water layer that could appear on the surface with a clear separation line between water and grout [4]. Bleeding tests were carried out in a graduated cylinder, where grout was placed, and the accumulation of bled water and the expansion volume were measured over 15, 30, 45, 60, 120 and 180 min. The tests were performed according to EN 447 and adapting of ASTM C940 [40,41]. Final bleeding (after 180 min) should be lower than 5%.
- Grouts must be suitable for injection through a syringe or tubing, to fill internal cracks and voids. An injectability test was carried out by injecting the grout at constant pressure to a vertically held column, from its bottom part (column was a transparent methacrylate tube height 390 mm and inner diameter 21 mm) (see experimental setup in Figure S1, Supplementary Materials). The column was filled with granular material whose characteristics are explained below (Table 3). This test is an adaptation of the sand column test (EN 1771—Determination of Injectability Using the Sand Column Test [42]), to be used for injection grouts. Injectability of a grouting mixture in a capillary network under predefined pressure is defined by the distance traveled by the grout as a function of time according to EN 1771. In this work, (according to the recommendations reported in Evaluation of Lime-Based Hydraulic Injection Grouts for the Conservation of Architectural Surfaces [43]), the material suggested in the standard for achieving a flow into a 0.2 mm crack in concrete is replaced by crushed travertine with grain sizes of 2–4 mm, a size that simulates an approximately 0.3–0.6 mm crack width. Each grout was prepared by mixing for exactly 3 min, using the same procedure adopted in the fluidity tests. The pressure used for filling the cylinders (0.075 MPa) was constant due to the use of an equipment of injection known as “pressure pot”, for 60 s. The time required for the complete filling of the cylinders was recorded.
  For these tests, high water/binder ratio of 1.24 was applied constantly due to the high water demand of the air lime and to the use of pozzolans [32,44].
  Several characteristics of the porous media were determined (Table 3): (a) parameters d(90) and d(10), which are respectively the diameter through which 90% and 10% of the total mass pass; (b) the total porosity, which was evaluated by measuring the volume of water which could be filled inside each cylinder, to know the available voids inside the column; and (c) the water absorption of the travertine.

During injectability tests, identical conditions were applied to the mixing procedure, and environmental conditions were kept constant. Pre-wetting was not applied due to the low water absorption of the travertine (6.6%) and to the detrimental effect on the adherence between the filler and the grout and on the mechanical strength reported [6].

Injectability rate was defined for numerical comparison, by using the time for the grout to reach the top of the cylinder, the quantity of injected grout, height of introduced grout and amount of the voids with the formula [6] given below:

$$I=\frac{1}{t}\cdot \frac{\frac{m}{\rho }}{V_{V}}$$

where I is the grout injectability (s^−1^), t the grout injection time to fill the injected height (s), m the injected mass during the injection process (g), *ρ* the density of grout (g/mL) and V_V_ is the voids volume of porous media (mL).

After the injectability experiments, the cylindrical methacrylate tubes with the fresh grouts were laid on a horizontal position and cured under lab conditions for at least 28 days. Slices extracted from the central part of the columns were cut, to assess the filling of the voids.

### 2.4. Hardened-State Tests

- Compressive strengths were measured after 7, 28, 91, 182 and 365 curing days in the 4 × 4 × 16 cm prismatic specimens. A device Proeti ETI 26.0052 (Proeti, Madrid, Spain) was used at a breaking speed 5–50 KP s^−1^ with a time interval between 30 and 90 s in the compressive strength tests.
- Thermal analysis of the hardened grouts was carried out with a simultaneous TG-sDTA 851 Mettler Toledo thermoanalyzer device (Schwerzenbach, Switzerland), using alumina crucibles. Samples were heated from 25 to 1000 °C, at a rate of 10 °C·min^−1^, under static air atmosphere.
- The porous structure of the hardened grouts was studied by Mercury Intrusion Porosimetry (MIP), using a Micromeritics AutoPore IV 9500 equipment (Micromeritics Instrument Corporation, Norcross, GA, USA) (pressure range 0.0015–207 MPa).
- The evaluation of the wettability of the hardened grouts was performed by measuring hydrophobicity through the static water contact angle of the samples, with an equipment OCA 15EC (DataPhysics Instruments GmbH, Filderstadt, Germany). Five water droplets at five different points of 5 μL were put onto the surface of the hardened grouts, and the reported results are averages of these measurements.

### 2.5. Durability

Prismatic specimens of the hardened grouts—prepared and cured 28 days as described before—were tested to assess the durability in the face of freezing–thawing cycles. The cycles for the evaluation of the frost resistance consisted of water immersion of the samples for 24 h and a subsequent freezing at −10 °C for 24 h. For these experiments, a CARAVELL 521-102 freezer (Caravell Ltd., Buckingham, UK) was used.

The structural integrity of the samples was visually assessed after the finishing of each freeze–thaw cycle, according to a previously reported criterion [22], which ascribes the following alteration state of the treated specimens:

- None: alteration for those samples with no evidence of decay.
- Scarce: for samples showing a slight degree of deterioration (some thin, short, shallow cracks on the surface of the specimens).
- Moderate: for altered samples, showing several deeper cracks.
- Large: for heavily altered specimens presenting deep cracks and a certain degree of swelling.
- Very large for samples with severe decay, large deep cracks, partial weight loss and large swelling.
- Total for destroyed samples, with only some parts remaining.

## 3. Results

### 3.1. Properties of the Fresh Grouts

#### 3.1.1. Fluidity (Spread Diameter)

**Figure 3:**
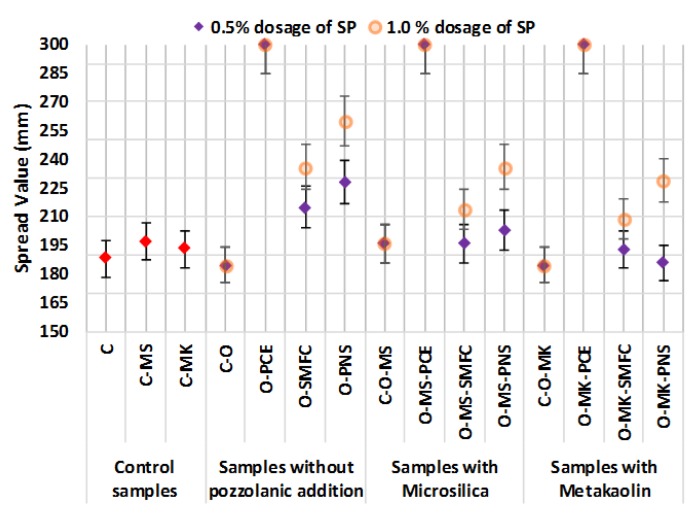
Spread diameter values of the different mixtures.

All mixtures without superplasticizers (control samples) presented similar fluidity values as compared to that of the control grouts (sample C), as measured by the spread diameter in the flow table test (Figure 3). The pozzolanic additions showed a gradual spread reduction of the fresh grouts, the pattern being: free-pozzolan > microsilica > metakaolin. This finding may be explained by considering the increased water demand of the samples with pozzolanic addition due to the high specific surface area of the pozzolans. This is in line with the observed spread diameter reduction in air lime pastes with pozzolanic agents [11]. Differences between the two pozzolanic additions can be ascribed to the different particle size. In spite of the relatively small surface area differences and to the similarities in reactivity and particle size reported in the literature for these additions [45,46], in the current work, as observed in Figure 2, MS particles exhibited a strong tendency to flocculate in the used aqueous systems, thus giving rise to large and less reactive agglomerates.

The influence of the superplasticizers on the spread values of fresh grouts with these admixtures was as follows. PCE addition resulted in a sharp fluidity increment, with spread diameter values higher than 300 mm, irrespective of the mix composition. Thus, PCE was seen to yield high-fluidity grouts, in good agreement with the previously reported effectiveness of the polycarboxylate ether derivatives both in lime and cement-based systems [11,14,26]. 

PNS and SMFC showed a similar behavior in all mixtures, for each one of the two tested dosages. The effectiveness of the dispersing action, as measured by the spread, was not as good for these SPs as it was for PCE. The similarity between PNS and SMFC arises from the likenesses in their linear molecular structure, in line with the general structure of these admixtures reported by Gelardi et al. [47]. These SPs exhibit mainly an electrostatic repulsion mechanism, due to their flat adsorption onto the binder particles and to their high anionic charge density (see Table 1), whereas steric hindrance was seen to play a minor role, as described by Pérez-Nicolás et al. [27]. This action mechanism has been proven to be less efficient than the steric hindrance action (prevalent for PCE). 

**Figure 4:**
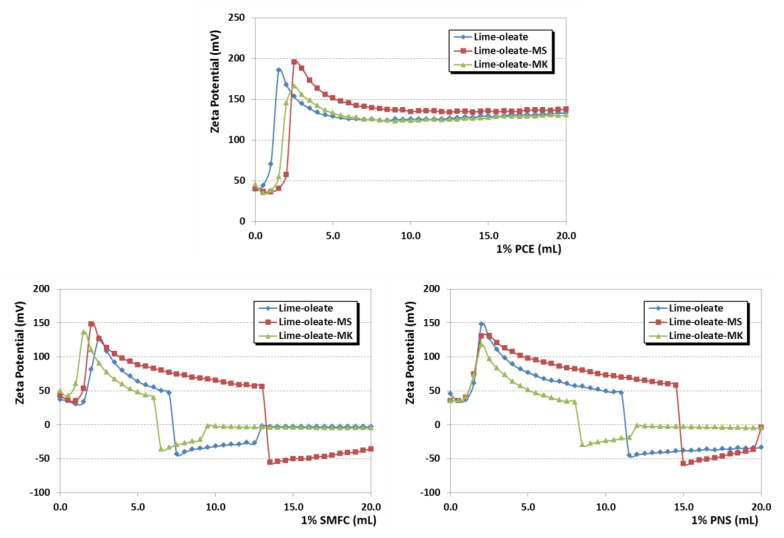
Zeta potential values of the binary lime + oleate and ternary lime + oleate + pozzolan systems titrated with the three SPs (PCE, SMFC and PNS).

Zeta potential measurements were carried out, upon titration with the different polymer-based SPs, for the lime–oleate pastes, as well as for the same pastes with the two pozzolanic additions (Figure 4).

As it can be seen, the pastes initially (before the addition of SPs) yielded positive zeta potential values (40–50 mV). Pérez-Nicolás et al. [27] indicated that air lime particles exhibited positive zeta potential values due to the positive charge of the portlandite crystals. In the presence of pozzolanic addition, the expected formation of C-S-H compounds has also been observed to yield high positive values of zeta potential: The negative charge caused by the silanol groups’ (Si-O-H) deprotonation in the C-S-H phases is strongly sheltered by the adsorption of the Ca^2+^ ions [27], recognized as potential-determining species [48], leading to a positive overcharging phenomenon.

Consistently, zeta potential values remained practically unaltered after the first 3 to 5 additions of SPs (Figure 4). After that, a dramatic increase in the zeta potential was observed irrespective of the SP tested, and then a gradual decrease toward lower zeta potential values was observed. The formation of a second adsorption layer accounts for this finding.

In the presence of the oleate chains, and owed to the addition of the SP, the adsorption saturation dosage of the first layer was quickly achieved, and a second layer of calcium ions sheltered the first polymer adsorption layer, thus resulting in a sharp increase of the zeta potential values. A second layer of adsorbed polymer started on top of the calcium ions’ layer, explaining the gradual decrease as a consequence of the negatively charged polymeric molecules (due to the deprotonation of the active groups at the alkaline pH) and of the displacement of the shear plane of the outer Helmholtz layer [11]. 

**Figure 5:**
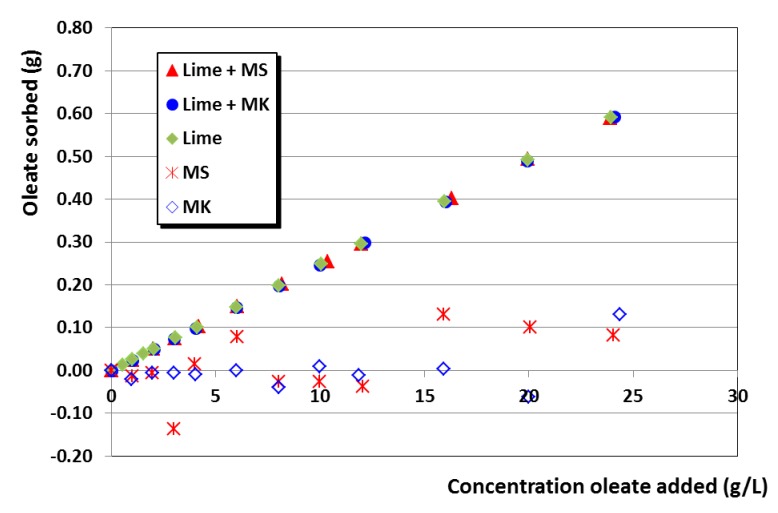
Adsorption isotherms of the oleate onto systems of lime, pozzolans and lime with pozzolanic additives (MK or MS).

**Figure 6:**
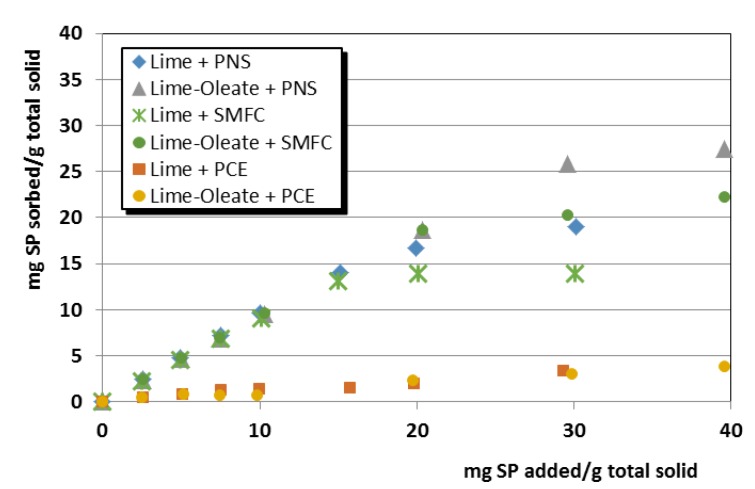
Adsorption isotherms of the three tested SPs onto systems of lime and lime with pre-adsorbed oleate.

**Figure 7:**
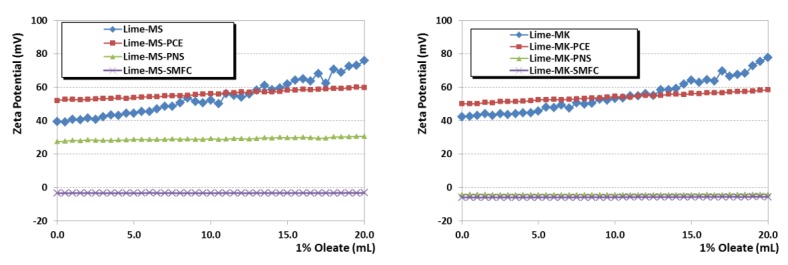
Zeta potential of simple lime systems, binary systems of lime with pozzolanic additives (MS, left diagram; MK, right diagram), and ternary systems with pozzolanic additives and pre-adsorbed SP. All the systems were titrated with a sodium oleate (1 wt. %) solution.

In support of these assertions, several experimental findings can be argued:

(i) Adsorption isotherms of sodium oleate onto lime particles (with and without pozzolanic additives) revealed a very strong adsorption of oleate onto these particles, making it reasonable achieving the saturation dosage of the first layer (Figure 5). Almost-negligible adsorption was observed for aqueous suspensions of pozzolans, confirming the strong influence of Ca^2+^ ions on the oleate adsorption, in agreement with the reported values in previous works by Wang, Z. et al. and Wang, Y. et al. [49,50] that described a sharp oleate adsorption onto minerals in the presence of calcium cations.
(ii) Adsorption isotherms of the superplasticizers onto lime particles, in which oleate was previously adsorbed, also showed the ability of the SPs to be adsorbed in a similar amount to the one that took place in the plain lime systems (Figure 6). This adsorption onto lime particles in which oleate molecules were pre-adsorbed can only be explained by assuming a double-layer adsorption. Isotherms also fit well into a Freundlich model (see high R^2^ values in Table 4).
(iii) Zeta potential curves obtained for lime systems (with or without pozzolanic agent) with pre-adsorbed superplasticizer, upon titration with a sodium oleate solution, were totally different (Figure 7): All curves showed a slight and continuous increase toward more positive values, without any sharp change in the curves. The zeta potential curves followed the same pattern as that of the SP-free systems titrated with sodium oleate. These curves could correspond to a simple monolayer adsorption process, in which oleate was adsorbed onto (a) free active sites and (b) in the sites previously occupied by SP molecules, which were removed due to a competition process.
  This assumption was later confirmed by adsorption isotherms studies of oleate in lime systems with pre-adsorbed superplasticizer. It was seen that all added oleate remained fully adsorbed, whereas the concentration of SP in the supernatant solution increased as more oleate was added. For example, this system yielded a 100.0 ± 0.9% of PCE in solution (that is, all the PCE was released), whereas the adsorption of PCE in a system with pre-adsorbed oleate resulted in a lower non-adsorbed polymer percentage of 92.94 ± 0.2% (that is, 7.06% of PCE remained adsorbed), thus confirming, as explained in (ii), the double-layer adsorption. This strong and competitive adsorption of oleate can be understood when considering its higher anionic charge density in comparison with the SPs.
(iv) Furthermore, the literature has described the multilayer adsorption of polymers onto mineral particles, as, for example, in the case of oleate (forming calcium dioleate layers) [50] and in the case of other superplasticizers [27,51].

Different patterns were observed in the zeta potential curves from the point of highest value onward, upon further additions of the three tested SPs, as can be seen in Figure 4:

(a) For PCE, zeta potential moved slightly toward lower positive values. The adsorption of the PCE (depicted in this second part of the curves of zeta potential) did not cause a substantial surface charge modification, confirming the weak influence of the anionic charge of this SP (which was the lowest, as reported in Table 1). The strong steric hindrance of the side chains of this polymer is more effective than the electrostatic repulsions of the negatively charged carboxylated groups. The predominant effect of the steric hindrance in this polymeric SP was confirmed by its high impact in fluidity (Figure 3), while it simultaneously did not dramatically modify the surface charge of the particles. The literature agrees about the prevalence of the steric hindrance mechanism for similar polymer molecules [51,52,53].
(b) For SMFC and PNS, the adsorption of the SP caused a clear decrease in the zeta potential values (sharper in the case of SMFC), finally resulting in a charge reversal into negative values of the zeta potential (Figure 4). The action mechanism of these two polymers can be linked to the electrostatic repulsions, particularly under alkaline conditions that fostered the ionization of the sulfonic groups [51,54]. The dosage at which the IEP was achieved would be the optimum dosage of the SP. SMFC inverted the sign of the surface charge at lower dosages and should be expected to be more effective than PNS. The higher molecular weight of this SMFC polymer (Table 1) contributes to enhance the predominantly efficient steric repulsions, thus explaining these experimental findings.

#### 3.1.2. Workability

**Figure 8:**
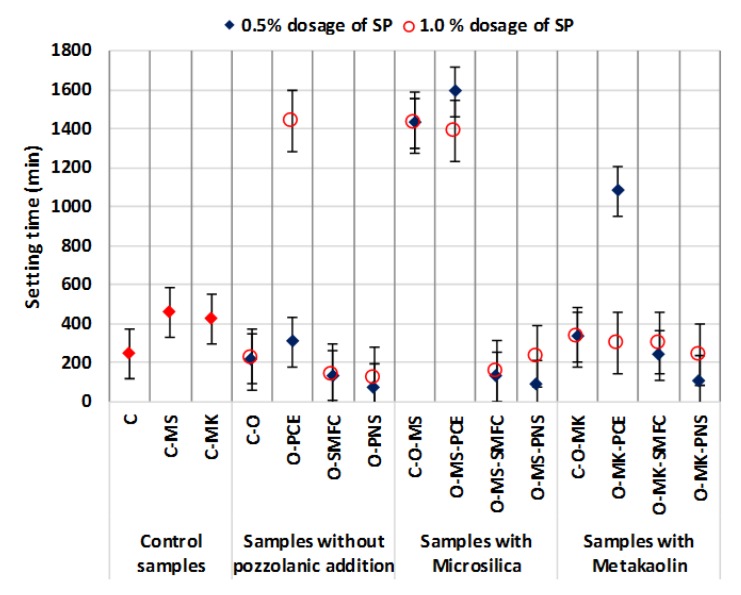
Workability of the different mixtures.

The use of PCE induced noticeable changes in workability for almost all mixtures (Figure 8). The addition of the PCE in microsilica-bearing samples gave rise to a delay in the stiffening of the grouts. This well-known effect has been pointed out in previous works and can be ascribed to the attachment of the PCE onto binding particles, which hinders their irreversible agglomeration, avoiding the early hardening of the grouts [11,55]. This delay was not observed for samples with MK at the highest dosage of PCE and can be explained by considering the fast pozzolanic reaction of the MK, as compared with microsilica. Stiffening time was shortened when SMFC and PNS were added as SPs. In almost all cases, for these two SPs (SMFC and PNS), the use of a 0.5% dosage yielded a sharper shortening of the stiffening time, which is directly linked to the poorer dispersing action of the lowest SP dosage due to the lower amount of adsorbed polymers.

#### 3.1.3. Bleeding

Bleeding of a fresh grout affects the quality of injection, since it causes clogging during application. A high bleeding value is an indicator of the absence of stability of the grout and can be due to the presence of admixtures of different hydrophilic character in the mixture. This segregation could increase along time, at least in the initial steps of the process. During the grout injection, bleeding undermines the effectiveness of the grout, because the upper part of the pores cannot be filled, due to the excess of water [4]. To assess the stability of the designed lime grouts, percentages of volumetric changes and bleeding of the different mixtures were determined (Table 5). All assayed samples presented very small volumetric changes, always below 1% (results not shown). The bleeding percentages were also low, and the obtained results fell within the tolerable limits (below the threshold value of 5%), as reported elsewhere [13,40].

Different percentages of segregation were obtained for each group of studied mortars. Samples without pozzolanic addition exhibited the lowest bleeding values. Samples with pozzolanic additions yielded higher bleeding values, although all of them were below the limit value. Accordingly, the designed grouts do not present excess of free water and can be considered as stable [12].

PCE as admixture with pozzolanic materials presented the lowest bleeding percentages, except for samples with microsilica. This fact can also be related to the setting time delay, since PCE hindered the irreversible agglomeration of the binding particles, thus causing segregation [11].

#### 3.1.4. Injectability

Water/binder ratio, the type and percentage of superplasticizer, the mixing procedure, grain size, pore size, total porosity and water absorption capacity of the filling material can be mentioned among the parameters influencing the quality of a grout injection [1,6,13,15]. 

Historic masonries were simulated by using methacrylate cylinders filled with travertine, in order to reproduce the inner part of historic walls. Cylinders were filled with the 2–4 mm fraction travertine type. With the aim of providing a reliable model of injectability, the use of just stable filling materials was selected rather than using filling material combinations. Furthermore, we did ascertain that filling particles had the same size distribution and their water absorption capacities were also the same to each other, avoiding variability due to the moisture content of the particles [1,56]. 

**Figure 9:**
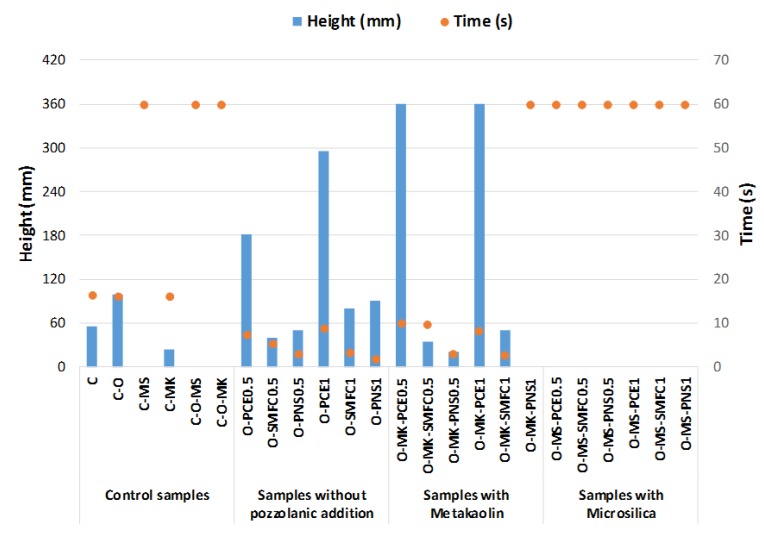
Injection height values and time of injection in the cylindrical columns for the different grouts.

The injectability was measured for the designed grouts, including the two dosages of the SPs. The time of filling and the height reached by the grouts, upward from the bottom of the cylinders, were measured and are displayed in Figure 9. The injectability (s^−1^) was calculated as detailed in Section 2.3 and values are collected in Table 5.

Grouts without microsilica addition (control samples) were able to flow through the column, at least partially. Plain lime grout (C) reached a height of 55 mm, and oleate–lime grout (C-O) went up to a height of 100 mm, both taking ca. 16 s in the excursion. Slump measurements showed very similar results for these two grouts, although injectability differs, possibly owing to the air-entraining action of the oleate, since small air bubbles could contribute to enhance the injectability, as previously reported [18].

The addition of pozzolans did complicate the injectability of grouts. The mixture C-MK reached 25 mm in 16 s, in good agreement with the fluidity observed during the flow table test (Figure 3). However, the simultaneous presence of oleate and metakaolin hindered the injectability of the grout. On the other hand, the addition of microsilica was fully detrimental for the injectability of the grouts. All the microsilica-containing mixtures, including the controls (C-MS and C-O-MS), were incapable of flowing through the column. The microsilica particles acted as a barrier, because of their well-known cohesive forces, preventing the grout injection [57]. This finding is in very good agreement with the large particle size of the microsilica agglomerates earlier measured (Figure 2), complicating the achievement of the necessary yield stress at the injection front and making it difficult to flow through the fine voids of the travertine particles filling the cylinder. In the end, this resulted in a blockage of the grout penetration and in an injectability obstruction [6]. Accordingly, the addition of a maximum 10% by weight dosage of this kind of pozzolanic materials is recommended to improve injectability and to increase mechanical strength in the hardened state [25].

The addition of the three tested polymeric superplasticizers resulted in different performances with respect to the injectability of the grouts. The simultaneous presence of sodium oleate appeared not to foster the injectability (as is the case for samples with PNS and SMFC in comparison with pure air lime grout control and oleate-containing control). This result is an expression of a certain incompatibility between the two admixtures (water-repellent and superplasticizer): As discussed previously, in Section 3.1.2, the strong adsorption of oleate onto lime particles restricts the attachment of the superplasticizers onto these particles, thus reducing the SP effectiveness. This fact seems to be of the utmost importance for the two superplasticizers whose action mechanism is mainly based on electrostatic repulsions (PNS and SMFC). The dosage of 1% (O-SMFC1 and O-PNS1) showed very similar values to those published by other authors (which were between 0.016 and 0.038 s^−1^) [1,6,12,44].

For PCE, its electrosteric repulsion mechanism allowed a better effectiveness, even with a reduced number of attached molecules of SP. In addition, the favorable plasticizing effect of non-adsorbed molecules of polycarboxylate ether derivatives, which remain in the interstitial solution, must be considered, in line with previous findings [58,59,60].

The addition of metakaolin depicted a very different performance pattern between the SPs, according to their respective main action mechanisms. The addition of MK worsened the injectability for samples with SMFC and PNS. These results are in line with those obtained from the flow table test and can be explained by considering the increase in the water demand due to the pozzolanic agent and the consumption of SP due to the progressive formation of C-S-H phases. The latter effect has been well described for cement-based materials in the case of flat polymeric admixtures [58,61].

Conversely, for grouts with PCE, the presence of MK enhanced the injectability, and the grout filled the whole column in a very short period (less than 10 s). The addition of a material with high surface area led to an increase in the anchorage active sites for PCE. The branched molecular architecture of this polymer, together with the recognized activity of the non-attached molecules, clearly improved the injectability of these PCE–MK grouts. The effectiveness of the dispersing action of polycarboxylate ethers in lime-based systems has been noticed in previous works [11,14,17,21] and confirmed in the current research. Grouts, including PCE as superplasticizer, exhibited the highest injectability values in all cases. The O-MK-PCE grout showed the best injectability, 0.08 s^−1^, which is larger than results reported by other authors [1,6,12,44].

In general, the increase in the dosage of the superplasticizer enhanced the grout injection. A dosage increase of up to 1% of PNS and SMFC in the C-O mixtures caused the rate of injectability to double (Table 5). Additionally, the low measured bleeding (below the limit of 5%) guaranteed the absence of the instability phenomena that are pointed out in the literature due to an excessive dosage of SP [6].

**Figure 10:**
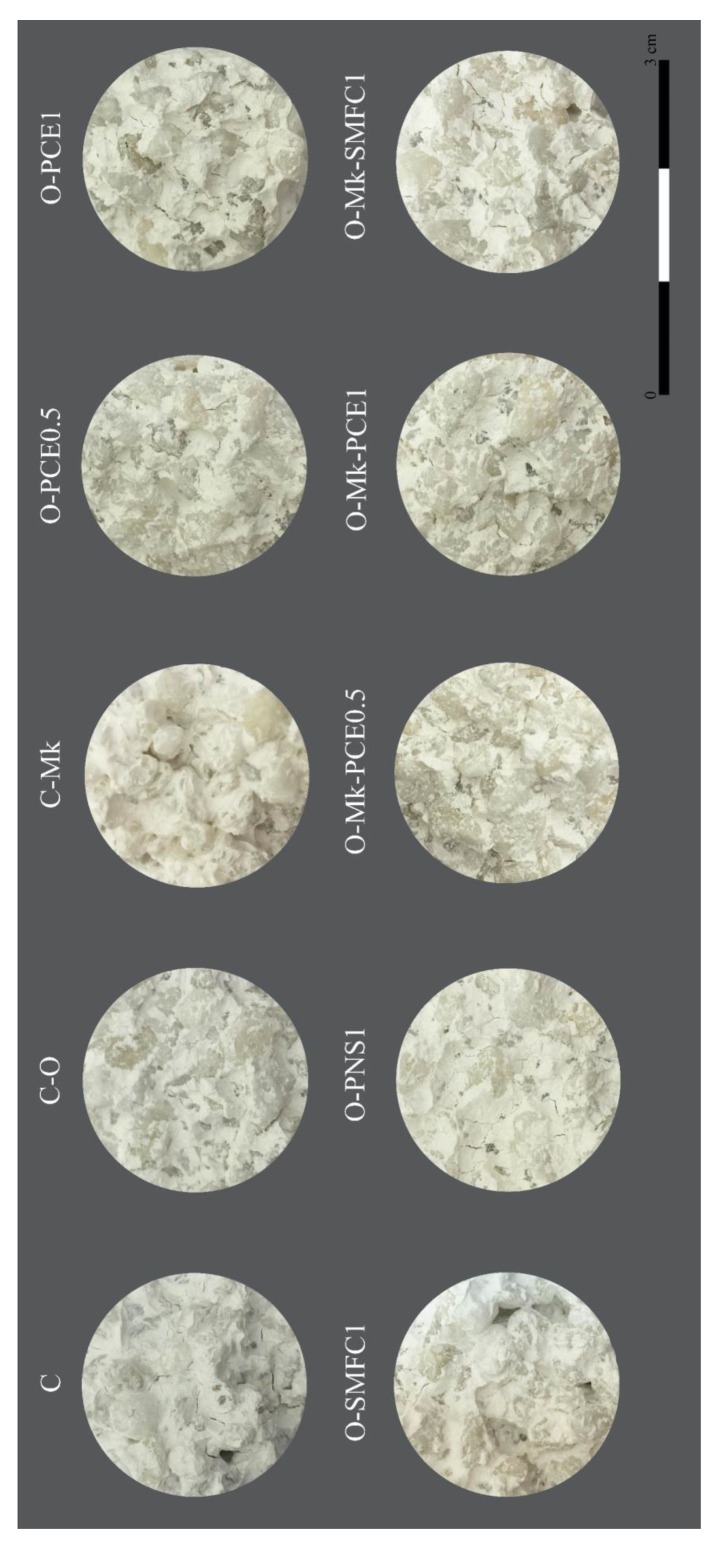
Section images of cylinders filled with grouts after 28 curing days.

After 28 curing days, slices from the central part of the columns were extracted and scrutinized, to assess the filling of the voids (Figure 10).

As shown in Figure 10, control grouts and grouts containing either PNS or SMFC were not able to fully fill the voids between the travertine fragments (slices showed holes and cracks, with heterogeneous areas caused by the poor injectability of these grouts and insufficient adherence to the travertine particles). Besides, C-MK sample showed a very poor wrapping of the travertine grains; holes are clearly visible in samples C, O-SMFC1 or O-MK-SMFC1; cracks are evident in samples C, O-PNS1 and O-MK-SMFC1.

On the contrary, grouts with PCE as superplasticizer evidenced good injectability, showing a complete filling of the voids and a good adherence to travertine grains. The grout O-MK-PCE depicted the most homogeneous aspect, proving the success of this injection grout in terms of penetration and diffusion. Solid images of the cylinders without holes were observed for this grout, implying that voids were successfully filled, thus resulting in a reinforcement of the masonry and hence of its strength.

### 3.2. Hardened Grout Properties

#### 3.2.1. Compressive Strength

**Figure 11:**
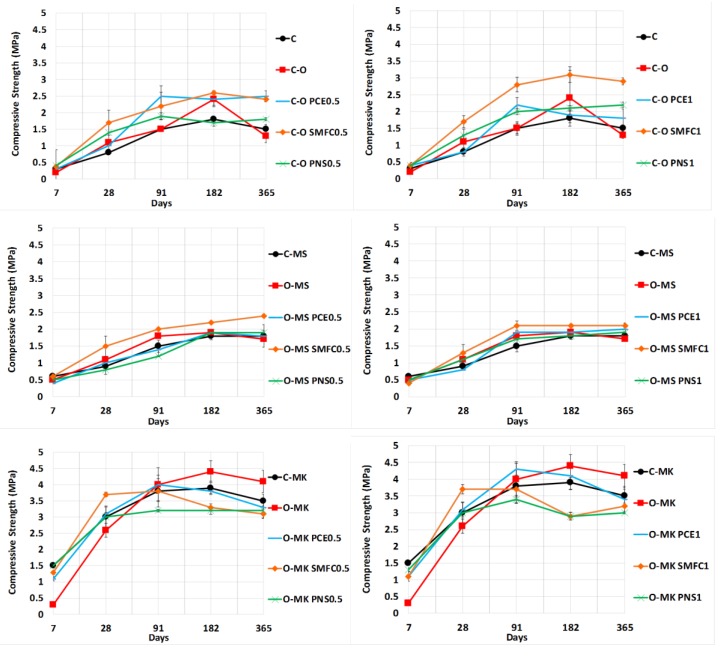
Compressive strength of grouts at different curing times (SP dosages: 0.5% and 1%).

During the hardening process, plain lime-based systems exhibited an increase in mechanical properties, thanks to the carbonation process in which CaCO_3_ is formed over time. Therefore, the values of compressive strength after long-term curing times—182 and 365 days—were greater on average (Figure 11). Pozzolanic reaction and formation of C-S-H phases in grouts with pozzolans also contributed to the strength of the hardened grouts.

**Figure 12:**
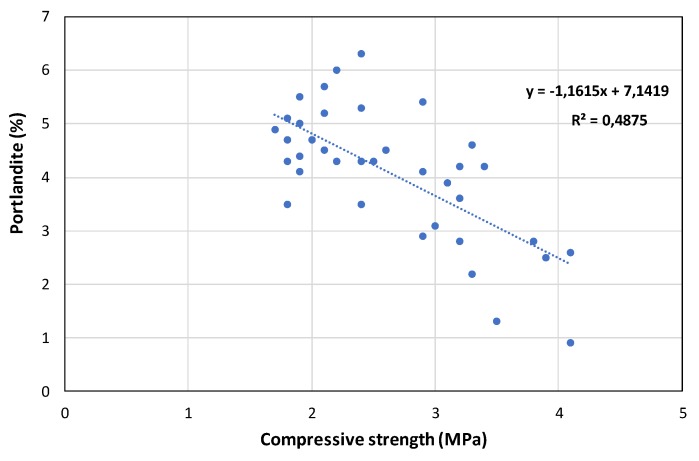
Correlation between compressive strength and portlandite (% calculated from TG results) in grouts after 182 and 365 curing days.

The addition of metakaolin increased the compressive strength, and the highest mechanical strength was obtained in the control mixture O-MK after 365 curing days (Figure 11). Microsilica was not so effective in increasing the strength. Its pozzolanic activity was lower as compared with MK, due to the larger particle size of the microsilica. TG-DTA analysis (Figure S2, Supplementary Materials) confirmed the differences: The percentages of Ca(OH)_2_ were lower for MK-bearing grouts (values after 182–365 curing days were on average below 4%, whereas samples with microsilica exhibited higher percentages of Ca(OH)_2_), suggesting a greater consumption of Ca(OH)_2_ during the pozzolanic reaction with MK. A Pearson correlation (coefficient 0.654, *p* < 0.01**) between the compressive strength and the percentage of portlandite was established: The higher the percentage of portlandite, the lower the compressive strength (Figure 12). It can be seen that most samples with percentages of portlandite below 4% yielded compressive strengths higher than 3 MPa, whereas samples with Ca(OH)_2_ percentages > 4%, in general, resulted in compressive strengths below 3 MPa.

**Figure 13:**
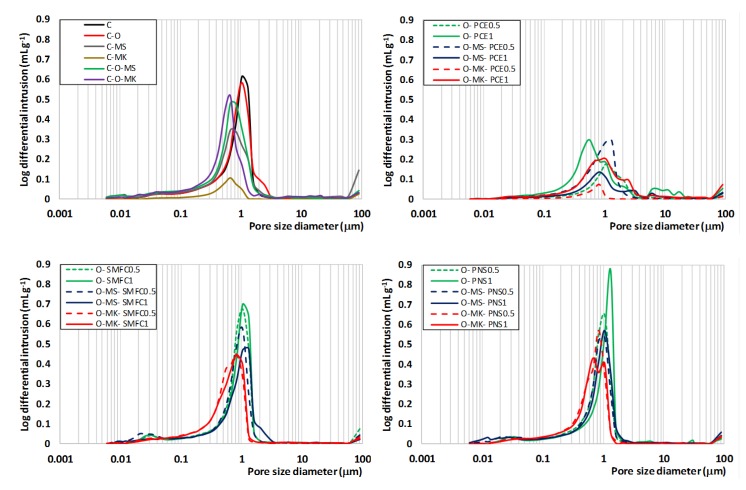
Pore size distributions of different paste samples after 365 days of curing.

The use of superplasticizers was, in general, favorable in order to increase the final mechanical strength of the grouts, PCE and SMFC, yielding the highest values. This finding is ascribed to the refinement of the pore structure caused by the superplasticizer, especially by PCE. The assessment of the pore size distributions (Figure 13) of the grouts showed the following:

- The addition of the pozzolanic additive (microsilica or metakaolin) reduced porosity by about 1 μm in diameter, due to the filling effect of the microsilica and the pozzolanic reaction (Figure 8, reduction in the area under the curve of the mercury differential intrusion).
- The addition of PCE caused a sharp drop in the number of pores, of about 1 μm. In addition, the main pore size shifted toward lower diameters (between 0.5 and 0.8 μm).

#### 3.2.2. Hydrophobicity

The static water contact angle and the time for the water-drop absorption are shown in Table 6. Measurements were carried out in grouts after 365 curing days. This parameter provides information about the real effect of the water-repellent agent, sodium oleate, and its compatibility with the SPs.

One of the intended characteristics of these grouts formulations was the hydrorepellency. The hydrorepellency is a superficial phenomenon caused by the tensioactive character of the sodium oleate. During the mixing process in an aqueous dispersion, the hydrophobic (non-polar) part of the molecule is oriented toward the aerial phase, whereas the polar segment is in the aqueous system. The combined effect of the pore size distribution (with small pores) and the active water-repellent agent led to suitable hydrorepellency. The O-MK-PCE1 grout exhibited the best hydrorepellency, thanks to its low total porosity (see Figure 13) and to the availability of molecules of the water-repellent agent (even assuming that most of the oleate molecules will be adsorbed onto lime particles). In this sense, previous discussions in Section 3.1.2, on the zeta potential values and on the adsorption of the SPs in the designed grouts, have shown the lowest interaction, and consequently the best compatibility, between the sodium oleate and the PCE. On the other hand, polymers SMFC and PNS yielded higher adsorption values onto lime particles, as reported in Figure 6.

#### 3.2.3. Durability

**Figure 14:**
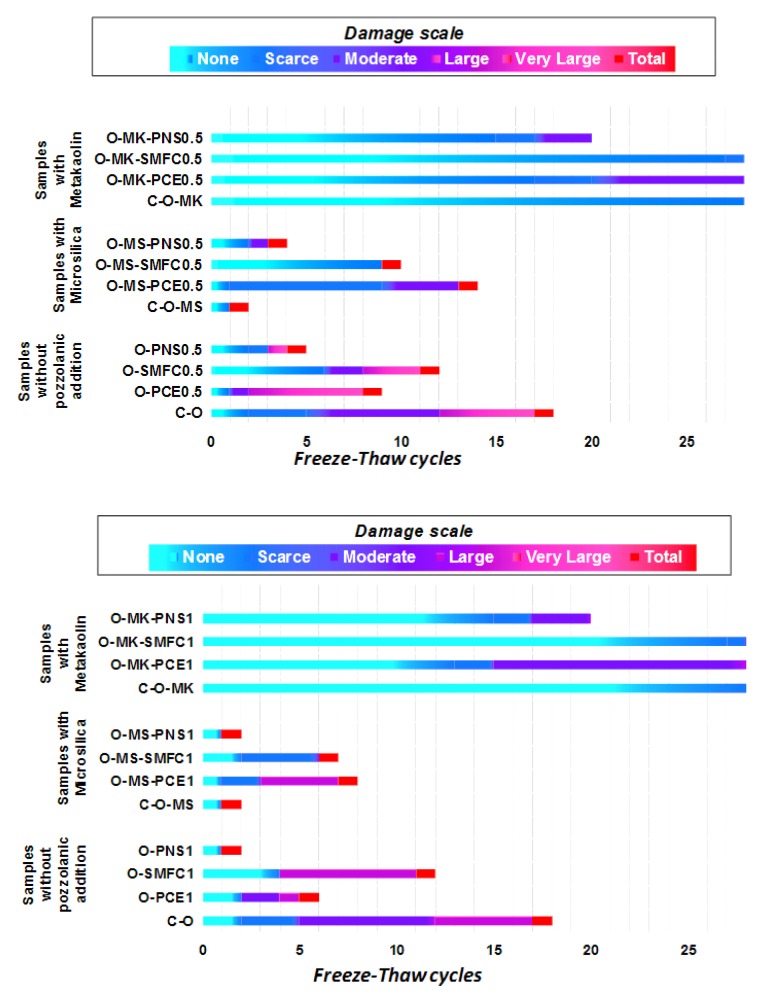
Values in the damage scale of grouts after freeze–thaw cycles.

The control grout formulations without a water-repellent agent were fully decayed after just one F–T cycle (total destruction of the specimens). The addition of sodium oleate definitely improved the freeze–thaw durability of the grouts, in accordance with our previous report on the positive effect of this admixture in lime-based mortars [18]. For instance, the control sample (C-O) resisted up to 18 freezing–thawing cycles (Figure 14).

The presence of pozzolanic admixtures in the grouts displayed different results: The addition of microsilica resulted in an adverse effect on the freeze–thaw durability, as can be seen in the control samples with microsilica (sample C-O-MS), showing serious decay after just two cycles (Figure 14). Conversely, the addition of metakaolin improved the resistance in the face of freezing–thawing cycles. According to the results, the reduction in the mean pore size prevented the absorption of liquid water, blocking its later freezing and expansion damage and thus providing better resistance against freezing–thawing cycles.

Metakaolin-containing samples showed a better durability when treated with SPs. Formulations with PCE and SMFC as SPs yielded the highest resistances.

## 4. Conclusions

Quaternary mixtures of air lime, polymer-based superplasticizers, a water-repellent agent and pozzolanic additives were studied as grouts to be used as repair materials for Built Heritage. The compatibility between the different admixtures was assessed.

Results showed that PCE was much more effective in increasing both the injectability and fluidity of the grouts than SMFC and PNS. The action mechanism of this polymeric superplasticizer was confirmed to be mainly steric, whereas SMFC and PNS acted through an electrostatic repulsion mechanism. The molecular architecture of these polymers was critical to explain their performance. 

Interactions with sodium oleate were found: The adsorption of sodium oleate onto lime particles was evident and caused a reduction in the superplasticizing effectiveness of the SPs, particularly of SMFC and PNS, as proved by the zeta potential measurements and adsorption isotherms. At the same time, the large adsorption of SMFC and PNS onto the oleate layer reduced the hydrorepellency of the treated grouts, as confirmed by the static water contact angle. The use of PCE was seen to be more favorable in terms of the highest injectability and hydrorepellency.

As for the pozzolanic additives, metakaolin imparted better characteristics to the grouts than microsilica, particularly in combination with SPs: higher injectability, better adherence and wrapping of the particles during injection, as well as higher mechanical strengths. Durability, in the face of freezing–thawing cycles, was also outstandingly increased due to the presence of MK. Microsilica showed a marked tendency to agglomerate in aqueous dispersions, which was strongly detrimental for the injectability of the grouts prepared with this pozzolanic additive. Besides, low mechanical strengths and poor durability were observed for grouts, including MS.

According to the results, the grout composed of air lime, metakaolin, sodium oleate and PCE, in its largest dosage of 1 wt.%, was found to be the most effective composition, improving the mechanical strength, the injectability and the hydrophobicity.

## Figures and Tables

**Table 1:** Characteristics of the polymers.

Admixture	Mw (Da)	Anionic Charge Density (meq g^−1^)	Elemental Composition					
			C (%)	H (%)	O (%)	N (%)	S (%)	Na (%)
PCE	8000	0.43 ± 0.05	47.62 ± 0.80	7.65 ± 0.13	42.2 ± 0.05	-	-	2.53 ± 0.01
SMCF	12,302	2.26 ± 0.04	20.80 ± 0.04	3.71 ± 0.05	31.83 ± 0.30	23.60 ± 0.24	10.7 ± 0.12	9.36 ± 0.20
PNS	8620	2.44 ± 0.07	43.92 ± 0.46	3.79 ± 0.01	29.03 ± 0.45	-	12.3 ± 0.19	10.96 ± 0.03
Oleate	n.d.*	3.32 ± 0.13	69.97 ± 0.03	10.50 ± 0.01	11.30 ± 0.20	-	-	8.30 ± 0.21

* Not determined.

**Table 2:** Composition of the grouts (% values).

	Name	Lime	Sand	Pozzolanic Addition *		Water- Repellent * *Oleate*	Superplasticizer *		
				*Microsilica*	*Metakaolin*		*PCE*	*SMFC*	*PNS*
Control samples (without polymeric superplasticizers)	C	25	75	-	-	-	-	-	-
	C-MS	25	75	20	-	-	-	-	-
	C-MK	25	75	-	20	-	-	-	-
	C-O	25	75	-	-	0.5	-	-	-
	C-O-MS	25	75	20	-	0.5	-	-	-
	C-O-MK	25	75	-	20	0.5	-	-	-
Samples without pozzolanic addition	O-PCE0.5	25	75	-	-	0.5	0.5	-	-
	O-SMFC0.5	25	75	-	-	0.5	-	0.5	-
	O-PNS0.5	25	75	-	-	0.5	-	-	0.5
	O-PCE1	25	75	-	-	0.5	1.0	-	-
	O-SMFC1	25	75	-	-	0.5	-	1.0	-
	O-PNS1	25	75	-	-	0.5	-	-	1.0
Samples with microsilica	O-MS-PCE0.5	25	75	20	-	0.5	0.5	-	-
	O-MS-SMFC0.5	25	75	20	-	0.5	-	0.5	-
	O-MS-PNS0.5	25	75	20	-	0.5	-	-	0.5
	O-MS-PCE1	25	75	20	-	0.5	1.0	-	-
	O-MS-SMFC1	25	75	20	-	0.5	-	1.0	-
	O-MS-PNS1	25	75	20	-	0.5	-	-	1.0
Samples with metakaolin	O-MK-PCE0.5	25	75	-	20	0.5	0.5	-	-
	O-MK-SMFC0.5	25	75	-	20	0.5	-	0.5	-
	O-MK-PNS0.5	25	75	-	20	0.5	-	-	0.5
	O-MK-PCE1	25	75	-	20	0.5	1.0	-	-
	O-MK-SMFC1	25	75	-	20	0.5	-	1.0	-
	O-MK-PNS1	25	75	-	20	0.5	-	-	1.0

* % by weight of lime.

**Table 3:** Porous media characteristics (travertine).

Characteristic	Value
d (90)	3.8 mm
d (10)	2.9 mm
Porous media porosity	47%
Water absorption	6.6%

**Table 4:** Parameters of the mathematical adjustment to Langmuir and Freundlich algorithms for the adsorption isotherms of SPs onto lime with pre-adsorbed oleate.

System	SP	Langmuir			Freundlich		
		qm (mg g^−1^)	b (dm^3^ mg^−1^)	R^2^	K (mg^1−1/n^dm^3/n^g^−1^)	1/n	R^2^
Lime–oleate	PCE	43.2	0.00001	0.1091	0.00186	0.8475	0.9485
Lime–oleate	SMFC	28.2	0.00026	0.9347	0.01615	0.8239	0.9763
Lime–oleate	PNS	36.8	0.00019	0.9056	0.02251	0.7790	0.9530

Notes: qm: maximum sorption capacity. b: the Langmuir constant. K, 1/n: the Freundlich constants. R^2^: correlation coefficient of the linear regression.

**Table 5:** Bleeding and injectability values (fresh grouts).

	Sample	Bleeding * *(%)*	Injectability *(s*^−1^*)*
Control samples (without polymeric superplasticizers)	C	-	0.006
	C-MS	<1%	0.016
	C-MK	<1%	0.000
	C-O	<1%	0.005
	C-O-MS	2	0.000
	C-O-MK	2	0.000
Samples without pozzolanic addition	O-PCE0.5	1	0.040
	O-SMFC0.5	<1%	0.015
	O-PNS0.5	2	0.022
	O-PCE1	2	0.050
	O-SMFC1	<1%	0.036
	O-PNS1	2	0.033
Samples with microsilica	O-MS-PCE0.5	<1%	0.000
	O-MS-SMFC0.5	<1%	0.000
	O-MS-PNS0.5	<1%	0.000
	O-MS-PCE1	4	0.000
	O-MS-SMFC1	2	0.000
	O-MS-PNS1	4	0.000
Samples with metakaolin	O-MK-PCE0.5	<1%	0.059
	O-MK-SMFC0.5	1	0.005
	O-MK-PNS0.5	2	0.010
	O-MK-PCE1	<1%	0.080
	O-MK-SMFC1	4	0.022
	O-MK-PNS1	4	0.000

* Values obtained according to EN 447 three hours after initial mixing.

**Table 6:** Results of the static water contact angle measurements (WCA) and of the time interval for the water-drop absorption.

	Sample	WCA	Time Interval for the Full Absorption of the Drop of Water		
			t < 5 s	5 s < t < 10 s	t > 10 s
Control samples (without polymeric superplasticizers)	C	-	⌧		
	C-MS	-	⌧		
	C-MK	-	⌧		
	C-O	84 ± 2.1			⌧
	C-O-MS	59 ± 2.1			⌧
	C-O-MK	35 ± 2.3	⌧		
Samples without pozzolanic addition	O-PCE0.5	70 ± 3.1	⌧		
	O-SMFC0.5	86 ± 2.6			⌧
	O-PNS0.5	105 ± 2.8			⌧
	O-PCE1	68 ± 2.1			⌧
	O-SMFC1	54 ± 2.9			⌧
	O-PNS1	40 ± 3.2	⌧		
Samples with microsilica	O-MS-PCE0.5	44 ± 2.5	⌧		
	O-MS-SMFC0.5	72 ± 2.2	⌧		
	O-MS-PNS0.5	83 ± 2.6			⌧
	O-MS-PCE1	98 ± 2.8			⌧
	O-MS-SMFC1	40 ± 2.4	⌧		
	O-MS-PNS1	37 ± 2.5		⌧	
Samples with metakaolin	O-MK-PCE0.5	44 ± 3.4	⌧		
	O-MK-SMFC0.5	112 ± 2.0			⌧
	O-MK-PNS0.5	89 ± 2.8	⌧		
	O-MK-PCE1	124 ± 2.5			⌧
	O-MK-SMFC1	44 ± 2.7		⌧	
	O-MK-PNS1	56 ± 2.4	⌧		

## Supplementary Materials

The following are available online at https://www.mdpi.com/2073-4360/12/4/887/s1, Figure S1: Setup of the injectability determination. Graduated methacrylate column filled with travertine porous medium; Figure S2: Percentages of portlandite (Ca(OH)_2_) of grouts at different curing times (TG results).
