# Supplementary material for: Combination of Polymeric Superplasticizers, Water Repellents and Pozzolanic Agents to Improve Air Lime-Based Grouts for Historic Masonry Repair
Source: Polymers (Basel). 2020 Apr 11;12(4):887. doi: 10.3390/polym12040887 (PMC7240504; doi:10.3390/polym12040887)
Supplement: Supplementary file 1 [file polymers-12-00887-s001.pdf]

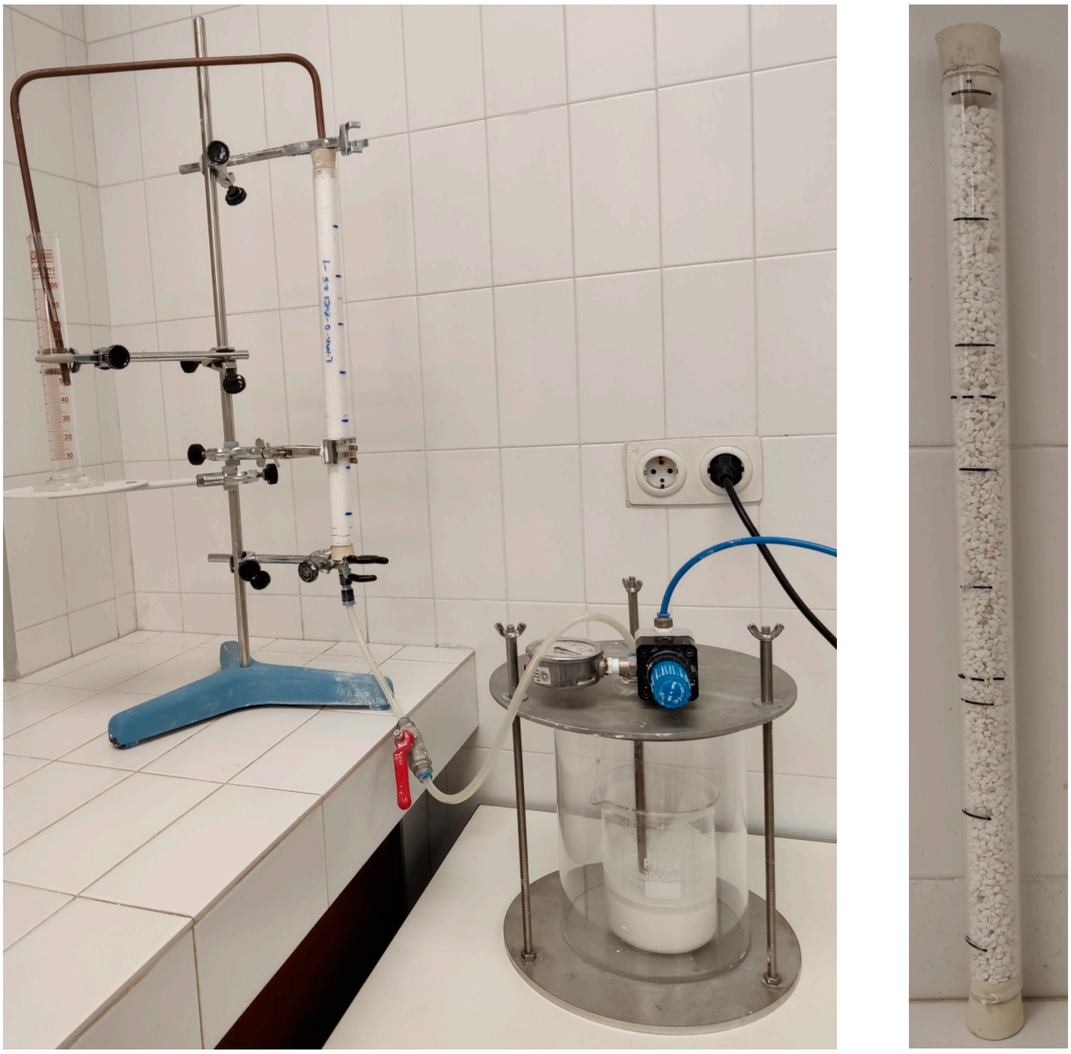

Fig. S1. Setup of the injectability determination. Graduated methacrylate column filled with travertine porous medium.

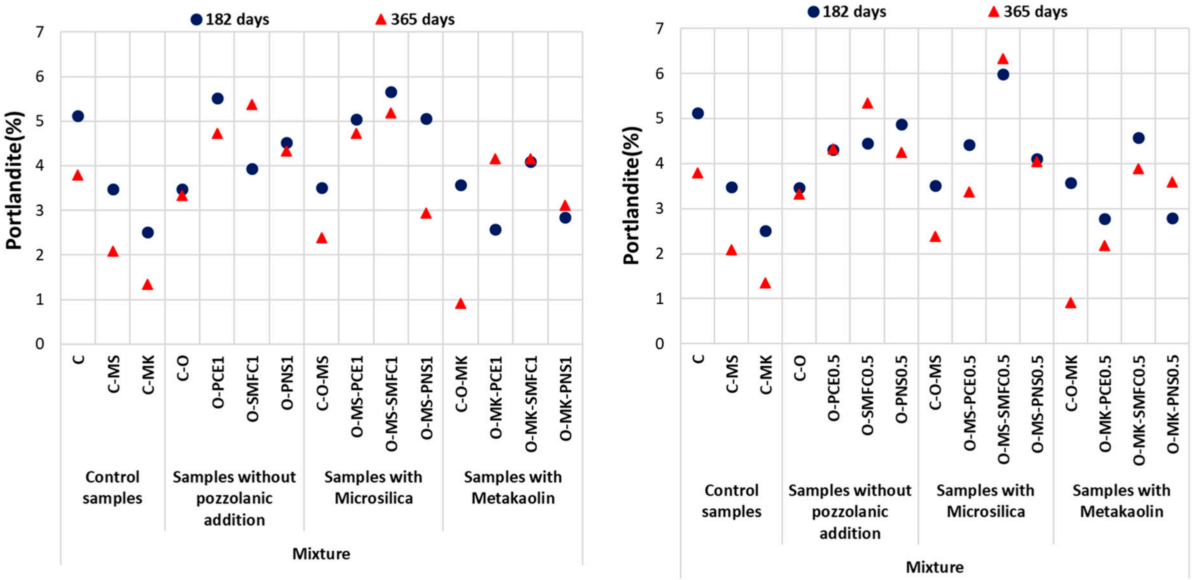

Fig. S2. Percentages of portlandite ($Ca(OH)_2$) of grouts at different curing times (TG results)
